# In Silico Drug Repurposing by Structural Alteration after Induced Fit: Discovery of a Candidate Agent for Recovery of Nucleotide Excision Repair in Xeroderma Pigmentosum Group D Mutant (R683W)

**DOI:** 10.3390/biomedicines9030249

**Published:** 2021-03-03

**Authors:** Yutaka Takaoka, Mika Ohta, Satoshi Tateishi, Aki Sugano, Eiji Nakano, Kenji Miura, Takashi Suzuki, Chikako Nishigori

**Affiliations:** 1 Division of Medical Informatics and Bioinformatics, Kobe University Graduate School of Medicine, Kobe 650-0017, Japan; mika@ebraille.med.kobe-u.ac.jp (M.O.); sugano@ebraille.med.kobe-u.ac.jp (A.S.); miuken@ebraille.med.kobe-u.ac.jp (K.M.); 0897vip@gmail.com (T.S.); 2 Department of Health Science, Kobe Tokiwa University, Kobe 653-0838, Japan; 3 Division of Dermatology, Kobe University Graduate School of Medicine, Kobe 650-0017, Japan; einakano@med.kobe-u.ac.jp (E.N.); chikako@med.kobe-u.ac.jp (C.N.); 4 Institute of Molecular Embryology and Genetics, Kumamoto University, Kumamoto 860-0811, Japan; tate@gpo.kumamoto-u.ac.jp

**Keywords:** drug repurposing, induced fit, molecular dynamics simulation, xeroderma pigmentosum complementation group D, ATP binding, 4E1RCat, nucleotide excision repair

## Abstract

Xeroderma pigmentosum complementation group D (XPD) is a UV-sensitive syndrome and a rare incurable genetic disease which is caused by the genetic mutation of the excision repair cross-complementation group 2 gene (*ERCC2*). Patients who harbor only XPD R683W mutant protein develop severe photosensitivity and progressive neurological symptoms. Cultured cells derived from patients with XPD (XPD R683W cells) demonstrate a reduced nucleotide excision repair (NER) ability. We hope to ameliorate clinical symptoms if we can identify candidate agents that would aid recovery of the cells’ NER ability. To investigate such candidates, we created in silico methods of drug repurposing (in silico DR), a strategy that utilizes the recovery of ATP-binding in the XPD R683W protein after the induced fit. We chose 4E1RCat and aprepitant as the candidates for our in silico DR, and evaluated them by using the UV-induced unscheduled DNA synthesis (UDS) assay to verify the recovery of NER in XPD R683W cells. UDS values of the cells improved about 1.4–1.7 times after 4E1RCat treatment compared with solvent-only controls; aprepitant showed no positive effect. In this study, therefore, we succeeded in finding the candidate agent 4E1RCat for XPD R683W. We also demonstrated that our in silico DR method is a cost-effective approach for drug candidate discovery.

## 1. Introduction

Xeroderma pigmentosum (XP), a UV-sensitive syndrome [1], is a rare autosomal recessive genetic disease with severe hypersensitivity to sunlight and neurological symptoms; no effective therapy is yet available. Neurological symptoms appear in 55% of all patients with XP before adolescence in Japan [2] and in 25% of patients with XP in Western countries [3]. XP occurs in 1 in about 22,000 people in Japan, 1 in about 1 million people in the United States, and 1 in about 435,000 people in Western Europe [4]. XP complementation group D (XPD) is the second most common complementation group in the United States and Europe and is the third most common group in Japan [3,5,6]. The XPD R683W mutant causes severe abnormalities of the central nervous system such as progressive intellectual impairment, hearing impairment, and loss of the ability to walk [7]. Because patients with the XPD R683W protein demonstrate reduced levels of nucleotide excision repair (NER) activity, we may be able to improve clinical symptoms if we could identify candidate agents to aid recovery of NER activity. XPD protein is an ATP-dependent DNA helicase in the transcription factor II human complex that causes a damaged DNA strand to unwind, which would facilitate DNA repair [8]. Our previous in silico study showed that clinical symptoms caused by an impaired NER function in each XPD type were determined by the ATP-binding ability in the XPD mutants [9]. This finding indicated that ATP-binding affinity may be used as a key indicator of drug discovery in silico. 

The usual model of drug development requires screening entries in a library of several million different compounds. In addition, evaluating the sensitivity and safety (toxicity) of the compounds and performing clinical trials are essential processes that take a long time and that must occur before new therapeutic agents are approved for clinical use [10]. These time-consuming barriers in the process of developing new drugs lead to the high costs associated with drug discovery. 

However, drug repurposing (DR) is an approach that may lower the costs of drug discovery and can be used with agents whose biokinetics and safety have already been confirmed by preclinical and/or clinical trials [11]. To overcome the difficulties in discovering new therapeutic drugs for rare diseases such as XPD, we describe, in this report, our new DR in silico strategy.

The key point of our new strategy is that the function of a target molecule (protein) is restored by induced fit, which changes the structure of the target molecule after ligand binding. The ligands used in our in silico DR strategy are compounds with previously confirmed biokinetics and safety profiles in preclinical and/or clinical trials, as just mentioned. The target molecule in this research was XPD R683W, which completely lacks the DNA repair function of XPD. Because we calculated the induced fit and reported the analytical speed of the calculation by using the K computer in a previous study [12], we included this calculation in our new in silico DR strategy.

In this research, we found, via our new strategy, candidate agents that restored the DNA repair function in silico. We then validated that these candidate agents restored the recovery of NER ability by using the EdU (5-ethynyl-2’-deoxyuridine)-based unscheduled DNA synthesis (UDS) assay [13]. This UDS assay showed that one candidate induced recovery of NER in XPD R683W primary cultured cells. This result suggests that our new in silico DR strategy—using a structural change in a molecule after induced fit—can assist in the identification of effective therapeutic agents. 

## 2. Experimental Section

**Figure 1:**
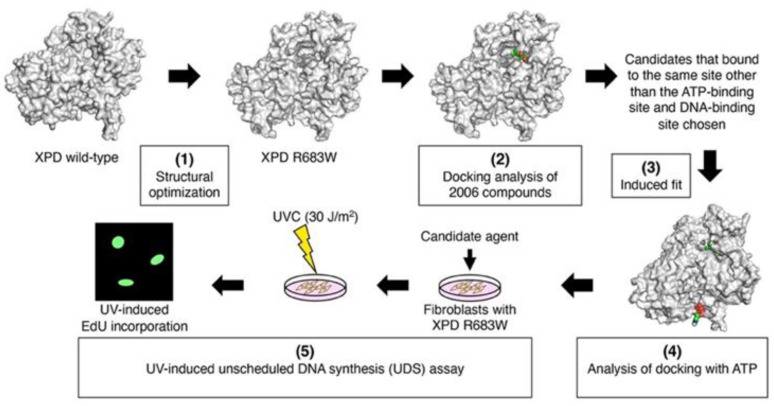
Schematic drawing of in silico drug repurposing (DR) and in vitro evaluations. The in silico DR procedure, by which we discovered therapeutic candidates, consisted of five steps: (1) analyzing the 3D structure of xeroderma pigmentosum complementation group D (XPD) R683W, (2) docking of XPD R683W with 2006 bioactive compounds, (3) performing induced fit with 5 complexes selected from 152 complexes, (4) evaluation of ATP docking with induced-fit XPD R683W, and (5) performing an in vitro DNA repair assay that used UV irradiation.

In this research, our new in silico DR strategy consisted of: (1) analyzing the three-dimensional (3D) structure of XPD R683W, (2) screening the compounds that we found in the bioactive compound library on the basis of the result of docking of the compound to XPD R683W, (3) optimizing the molecular structure for the induced fit of the complexes of XPD R683W with each compound, and (4) ATP docking to the induced-fit XPD R683W. Finally, we chose candidate agents on the basis of the result of ATP docking and the electrostatic surface potentials. Figure 1 illustrates these steps in our in silico DR strategy and in vitro validation.

### 2.1. Preparation of Compounds for DR and Construction of the 3D Structure of XPD R683W

We used water, salts, and counterions in bioactive compound structures of the Bioactive Compound Library (Selleck Chemicals, Houston, TX, USA) that we had obtained for studies of therapeutic agents for in silico DR. We then filtered 7072 bioactive compound structures to remove duplicates, after which we used molecular operating environment software (Chemical Computing Group, Montreal, QC, Canada) to subject the resulting 2006 compounds to energy minimization with the Merck Molecular Force Field MMFF94x until the RMS gradient value became smaller than 0.01 kcal/(mol·Å). We obtained the 3D structure of wild-type XPD from ModBase (Model ID: 44e58cd7effa445a0bb61e5c2f7fbf13). We then added hydrogen atoms, via PyMOL 2.0.6 software [14], to the model structure of XPD R683W after amino acid substitution by using molecular operating environment software.

We optimized the structural data by using GROMACS 4.6.5 software [15] on the K computer with the AMBER99 force field. We used the TIP3P three-site rigid water model [16] to solvate the protein. The minimal distance of a protein atom to the edge of the rectangular water box was 14 Å. We added Na^+^ and Cl^−^ ions to keep the whole system neutral, which led to salt concentrations of 0.15 M. We minimized the energy by using the steepest descent method until the maximum force on any atom was less than 1000 kJ/(mol·Å), which was followed by a 250 ps heating process to reach a 310 K starting temperature. After the heating process, we used a 5000 ps production run with the NPT ensemble (a constant number of particles (N), pressure (P), and temperature (T)). We maintained the temperature and pressure of the system by using the Berendsen coupling algorithm [17]. All bond lengths involving hydrogen atoms were constrained with the LINCS algorithm [18].

### 2.2. Docking Analysis of Compounds with XPD R683W and Optimizing the Structures of the Complexes

We used molecular operating environment software to analyze docking of the compounds with XPD R683W as follows: the entire molecular surface of XPD was defined as a docking site; five docking runs per compound were performed; the Triangle Matcher placement method was used for docking analysis; and all hydrogen atoms were added and water molecules were removed. After the docking analysis with XPD R683W, we chose 152 complex molecules; of these 152 molecules, 5 bound to the same site other than the ATP-binding site and DNA-binding site. We then used the London ΔG scoring function to optimize the structure (induced fit) with GROMACS software [15], and we chose the complexes that had the most stable energy (with 4E1RCat, aprepitant, ABT-737, bromosporine, and tanespimycin (17-AAG)).

### 2.3. Validation of Structural Optimization

We utilized the root-mean-square deviation (RMSD) and the Ramachandran plot to verify the accuracy of the modeled or induced-fit structures. We analyzed the RMSD from the 5000 ps molecular dynamics trajectory by using the g_rms GROMACS inbuilt tool, and we determined the quality of the 3D structures by using the PROCHECK program [19] for the Ramachandran plot.

### 2.4. Evaluation of the Recovery of ATP-Binding Activity

After the induced fit, we analyzed the docking with ATP via molecular operating environment software under two different conditions: 100 docking runs per candidate agent with the entire molecular surface of XPD defined as the docking site to show the ratio for the binding site; 10 docking runs per candidate agent with the ATP-binding site [20] defined as the docking site to show the ratio of the correct numbers of bindings [21]. We selected as therapeutic candidates the agents that increased the numbers of ATP molecules in the binding sites and the numbers of correct ATP bindings, and we analyzed these candidates in vitro. We also analyzed electrostatic surface potentials by using the Adaptive Poisson–Boltzmann Solver program [22].

### 2.5. Evaluation of the Agents In Vitro: UV-Induced UDS Measurement in Primery Human Fibroblasts by Using EdU Incorporation

The Medical Ethics Committee of Kobe University approved this work, which was conducted according to the Declaration of Helsinki principles. All patients provided written informed consent for participation in this study. We obtained primary cultured fibroblasts from skin biopsy specimens obtained from patients with XPD R683W who had homozygous mutations in the excision repair cross-complementation group 2 (*ERCC2*) gene [9]. All cultured cells were maintained at 37 °C in 5% CO_2_ in Dulbecco’s modified Eagle medium supplemented with 10% fetal bovine serum and penicillin-streptomycin-amphotericin B suspension (FUJIFILM, Tokyo, Japan). 

On the basis of the in silico results, we chose the candidate drugs 4E1RCat and aprepitant (Namiki Shoji Co., Ltd., Tokyo, Japan), dissolved in dimethyl sulfoxide, to treat cells derived from patients with XPD R683W. According to a previous report in which 4E1RCat showed an inhibitory effect on the start of cap-dependent translation at the 50 μM concentration [23], we performed UV-induced UDS assays at the following concentrations: 1, 10, 25, and 50 μM. Aprepitant had inhibited substance P at 1 nM [24], and the half-maximal inhibitory concentration of various cell lines was 20-30 μM [25]. Therefore, 1 nM, 1 μM, and 10 μM aprepitant concentrations were used for UDS tests.

To evaluate the effectiveness of candidate drugs on NER recovery, we performed the EdU-based UDS assay by using XPD R683W cells irradiated with ultraviolet C radiation (UVC), as described previously [13,26]. The UV source was Germicidal lamps emitting predominantly 254 nm light (Toshiba GL10, Toshiba Electric, Yokosuka, Japan). For the EdU-based UDS assay, 8 × 10^4^ cells were plated in 35 × 10 mm cell culture dishes (FALCON, Becton, Dickinson and Company, NJ, USA). After 48 h, drug candidates were added to each dish. Then, 24 h later, cells were irradiated with UVC (30 J/m^2^) [27] and were cultured for 2.5 h in 10 μM EdU. After that, cells were analyzed via Invitrogen Click-iT Plus EdU Alexa Fluor Proliferation Assays (Thermo Fisher Scientific, Inc., Rockford, IL, USA) to visualize EdU incorporated into genomic DNA in the cells. We analyzed the fluorescence of the nuclei in these cells and quantified the fluorescence by using the ImageJ program (Rasband, WS, NIH, Bethesda, MD, USA) [28] after photography via fluorescence microscopy (BZ-X710; KEYENCE, Hyogo, Japan). The UDS value of XPD R683W cells was expressed as relative to the UDS value of normal human primary cells as described in previous reports [27].

### 2.6. Statistical Analysis

Data are presented as means ± SD and were analyzed by using one-way ANOVA with Tukey’s post hoc test for comparison of UDS with R software (R Foundation, Vienna, Austria). A *p* value of < 0.05 was considered statistically significant.

## 3. Results

### 3.1. Validation of Structural Optimization

**Figure 2:**
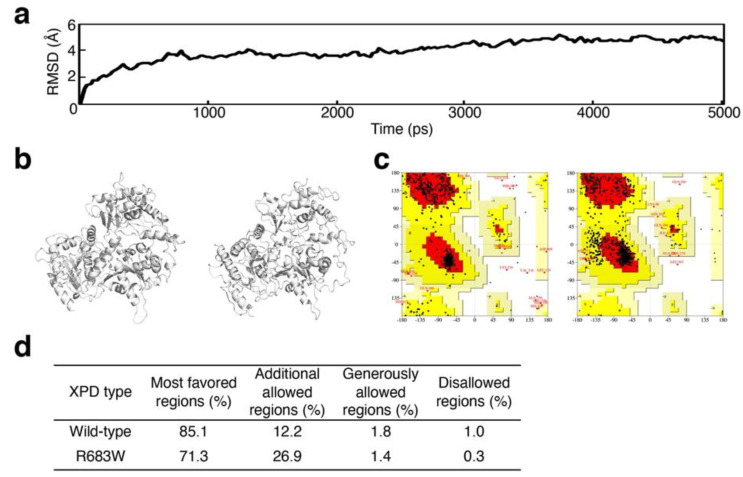
Validation of the 3D structures of wild-type XPD and XPD R683W. (**a**) The root-mean-square deviation (RMSD) for the backbone atoms of XPD R683W is shown as a function of time. (**b**) Cartoon representations of 3D structures of wild-type XPD (left) and XPD R683W (right). (**c**) Ramachandran plots for wild-type XPD (left) and XPD R683W (right). (**d**) Plot statistics for each XPD type.

In the molecular dynamics simulation analysis of XPD R683W, convergence was found based on the result that the trajectory was stable after 3500 ps (Figure 2a). With regard to the accuracy of the wild-type XPD and R683W XPD structures (Figure 2b), as analyzed via the Ramachandran plot in PROCHECK [19], both structures were adequate on the basis of 1.0% or less in the disallowed regions, as reported previously [29] (Figure 2c,d).

**Figure 3:**
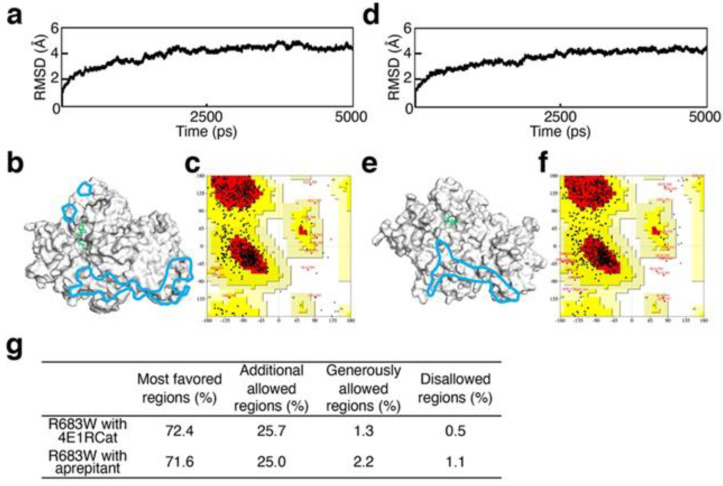
Validation of structural optimization. (**a**,**d**) The RMSD for the backbone atoms of XPD R683W with 4E1RCat (**a**) or aprepitant (**d**). (**b**,**e**) Cartoon representations of 3D structures of XPD R683W with 4E1RCat (**b**) or aprepitant (**e**). Blue areas indicate DNA-binding regions and green stick models indicate 4E1RCat (**b**) or aprepitant (**e**). ATP-binding regions are at opposite sides of these pictures. (**c**,**f**) Ramachandran plots for XPD R683W with 4E1RCat (**c**) or aprepitant (**f**). (**g**) Plot statistics for each complex of XPD R683W with the drug candidates.

Convergence of the 3D structures after induced fit was demonstrated by the stable trajectories after 3000 ps in all five complexes that we evaluated, which consisted of XPD R683 complexed with each candidate agent, as Table 1 shows. For example, Figure 3a,b shows the convergence result of the complexes with candidate agents chosen for in vitro analyses described in the next section. With regard to the accuracy of the induced-fit structures (Figure 3b,e) on the Ramachandran plots (Figure 3c,f), both were adequate on the basis of the 1.1% or less in the disallowed regions (Figure 3g).

### 3.2. Analysis of Recovery of ATP-Binding Activity In Silico

**Figure 4:**
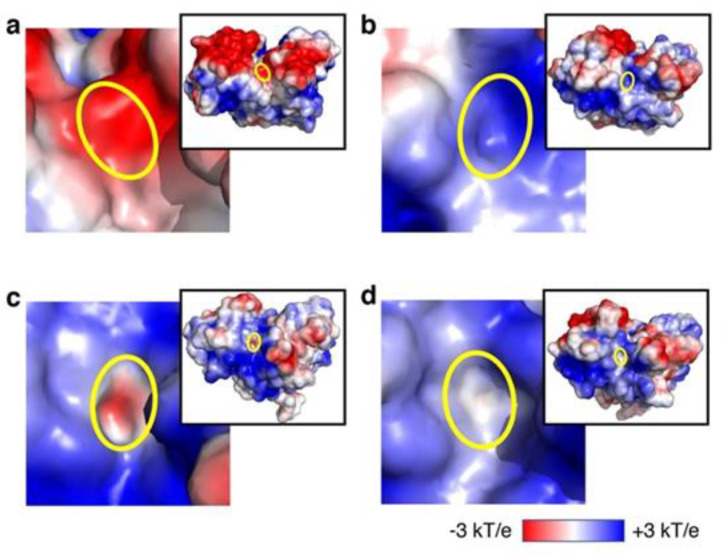
Electrostatic surface potentials of various XPD types. ATP-binding regions are outlined in yellow in wild-type XPD (**a**), XPD R683W (**b**), XPD R683W with 4E1RCat (**c**), and XPD R683W with aprepitant (**d**). (**c**,**d**) Effects of the candidate drugs on electrostatic surface potentials in ATP-binding regions of XPD R683W.

To study the effects of candidate agents on ATP binding to XPD R683W, we used ATP docking to XPD R683W after induced fit. After the docking, we compared our XPD R683W results with results for wild-type XPD without the candidate agents (Table 1), after which we performed in vitro analyses of 4E1RCat and aprepitant. We chose these two candidates because of the number of ATP docking occurrences in the correct binding mode (per 10 runs with ATP-binding region) and in the correct binding site (per 100 docking runs with entire the protein surface), if we assume that results for the wild-type were correct. The electrostatic surface potential of the ATP-binding region in XPD R683W shifted to a positive charge, in contrast to the negative charge in the wild-type XPD (Figure 4a,b). After the induced fit caused by the binding of XPD R683W to 4E1RCat or aprepitant, electrostatic surface potentials of the ATP-binding sites changed from a positive charge in XPD R683W to a negative charge, similar to the negative charge for wild-type XPD (Figure 4c,d). 

### 3.3. Effect of Candidate Agents on NER Recovery as Determined via the EdU-Based UDS Assay

**Figure 5:**
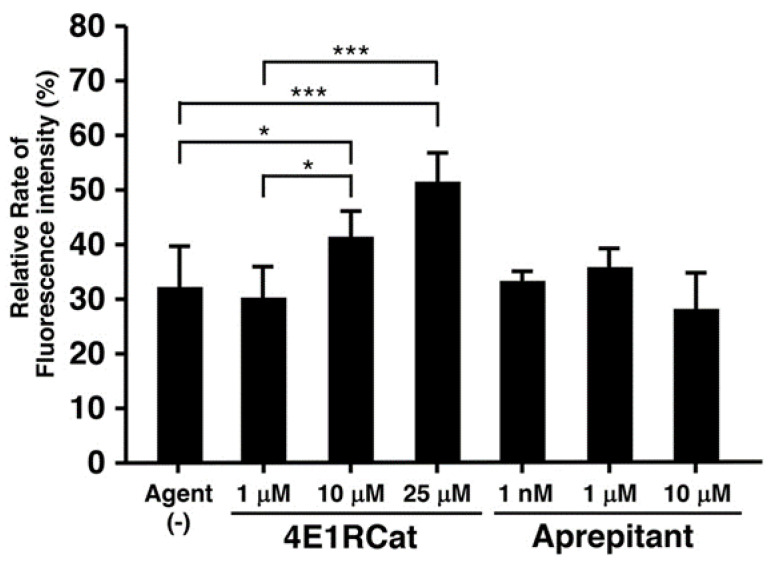
Recovery of nucleotide excision repair (NER) in XPD R683W cells. Two candidate agents chosen in silico, 4E1RCat and aprepitant, were evaluated via the EdU-based unscheduled DNA synthesis (UDS) assay. Data represent means ± SD. Statistical analysis was performed by using one-way ANOVA, and significance was defined as: * *p* < 0.05, *** *p* < 0.001 (Agent (-) vs. each concentration of agent).

To evaluate the efficacy of the candidate agents on NER recovery in XPD R683W cells, we subjected 4E1RCat and aprepitant, which we chose from the in silico results, to the EdU-based UDS assay via XPD R683W cells after UV exposure. Figure 5 shows the influence of candidate agents on UDS. 

UDS in a control experiment showed a value of about 30% compared with the normal cell control group (Figure 5, bar 1). UDS in the experiment in which a final 4E1RCat concentration of 10 or 25 µM was added to XPD R683W cells showed a value about 1.4 or 1.7 times higher than that in the solvent-only control group, respectively (Figure 5, bars 3, 4). However, no data were obtained for 50 μM 4E1RCat because artifactual fluorescence staining affected the whole XPD cell. Aprepitant treatment caused no NER difference (Figure 5, bars 5–7), and 30 μM aprepitant was cytotoxic (data not shown). 

## 4. Discussion

In this study, we showed that our in silico procedure can benefit new drug discovery via DR. The RMSD and Ramachandran plot statistics indicated the accuracy of each XPD structure evaluated (Figure 2 and Figure 3). On the basis of our previous report [9], we selected the candidate agents 4E1RCat and aprepitant because of the recovery of ATP-binding ability in silico. Their characteristics are as follows: 4E1RCat inhibits interactions between the eukaryotic initiation factors (eIF) 4E and G and reverses chemoresistance in anticancer therapy [23]; aprepitant is a selective high-affinity antagonist of human substance P/neurokinin-1 receptors as the inhibitor of substance P [24,25]. During our in vitro evaluation of NER recovery via the EdU-based UDS assay, 4E1RCat demonstrated a recovery of UDS in the primary cultured cells harboring the XPD R683W mutation, which indicated that the drug-induced recovery of NER in the cells (Figure 5). This recovery of NER may benefit therapeutic drug use in clinical settings because NER deficiency causes XPD pathogenesis. The mechanism for the recovery of NER in the cells by use of 4E1RCat may be explained by improved ATP binding (Table 1), which resulted because of the shift in charge from positive (Figure 4b) to negative (Figure 4c) in the ATP-binding region of the XPD R683W protein after 4E1RCat binding. However, only a slight difference occurred in the electrostatic surface potentials at the DNA-binding regions. With regard to aprepitant, no NER recovery was observed. This finding may be explained by the lack of effect on the charge status in the ATP-binding region in XPD R683W after binding with aprepitant (Figure 4d).

4E1RCat was first reported to be an inhibitor of eIF4E/eIF4G interaction and eIF4E/4E-BP1 interaction as determined from screenings of 217,341 compounds by using a time-resolved Förster resonance energy transfer-based assay [23]. In vitro investigations showed that 4E1RCat also halted the decrease in interleukin-1β production, myeloperoxidase levels, and neutrophil recruitment in murine *Aspergillus fumigatus keratitis* by means of inhibiting eIF4E/4E-BP1 binding, as well as stopping the increase in fungal load and apoptosis [30]. Another report suggested that 4E1RCat lowered interferon-α and interferon-λ levels in plasmacytoid dendritic cells by suppressing interferon regulatory factor 7 protein expression [31]. 4E1RCat can inhibit virus replication in cultured cells infected with coronavirus or Crimean-Congo hemorrhagic fever virus as a result of the inhibition of protein translation [32,33]. In animal research, 4E1RCat prevented upregulation of fibronectin and Nr4a and delayed cartilage degeneration in a rat osteoarthritis model after an intra-articular injection [34]; it also regulated obesity via inhibition of protein tyrosine phosphatase 1B activity (US Patent No. 2006/070135 A1). With regard to toxicity, 4E1RCat demonstrated no acute toxicity in mice given 15 mg/kg/day for 5 days by intraperitoneal injection [23]. Additional pharmacokinetic analyses and preclinical studies of animal models of XPD R683W are needed before human clinical trials can begin. 

To establish a new strategy of discovering candidate therapeutic agents by means of in silico DR, we subjected five compounds to structural optimization (induced fit) via the K computer. By means of our in silico analysis of these five compounds from the Bioactive Compound Library, we discovered one candidate agent—4E1RCat—and we determined the recovery of NER function by using cultured fibroblasts obtained from skin biopsy specimens. These findings suggest that our procedure is applicable to the discovery of new candidate drugs. However, additional in silico DR analysis is needed to confirm that this discovery is reproducible, not accidental. Moreover, additional candidate therapeutic agents for this disorder are required for the identification of effective therapeutic drugs. 

For the final determination of a therapeutic drug for this disease, additional in vitro studies of NER and use of an animal model of XPD R683W are needed. To accomplish this goal, we are planning to analyze ATP docking of XPD R683W to other candidate compounds after induced fit. In the future, additional therapeutic drug candidates may be discovered by using this in silico DR procedure. 

## Figures and Tables

**Table 1:** Results of docking simulation analyses of XPD R683W with candidate agents, and ATP docking to induced-fit XPD R683W.

Candidate Agents Tested with R683W	Correct Binding of ATP (per 10 Runs)		ATP in the Binding Region (per 100 Runs)		Docing Scores of XPD R683W and Agents
	*n*	Docking Score	*n*	Docking Score	
Wild-type (no agent)	6	−5.59 ± 0.35	1	−4.08	NA
4E1RCat	8	−6.26 ± 0.72	2	−6.22 ± 0.49	−6.80 ± 2.17
Aprepitant	4	−5.16 ± 0.70	1	−6.18	−6.35 ± 0.86
ABT-737	4	−6.44 ± 0.56	0	NA	−8.36 ± 1.39
Bromosporine	3	−5.87 ± 0.55	1	4.82	−6.43 ± 1.55
17-AAG	3	−4.96 ± 3.07	0	NA	−6.42 ± 1.10

Data are presented as means ± SD. A lower docking score indicates a more stable binding.

## Data Availability

Data sharing not applicable.
